# Supplementary material for: Investigating the impact of psychedelic drugs on social cognition defects: A scoping review protocol
Source: PLoS One. 2024 Jul 29;19(7):e0307491. doi: 10.1371/journal.pone.0307491 (PMC11285908; doi:10.1371/journal.pone.0307491)
Supplement: S1 Data — (PDF) [file pone.0307491.s002.pdf]

Data Extraction Form_Psychedelics for Social Cognition

Data Extraction Form-Psychedelics for Social Cognition

1. Title of Study

2. First Author (last name, first name)

3. Year of Publication

4. Study Design

5. Main Research Question

6. Aims of Study

7. Health Status of Sample (including any disorders experienced by the sample population)

8. Age of Sample

9. Sex of Sample

10. Treatment Administered

11. Dosage of Treatment Administered

12. Duration of Treatment

13. Setting Treatment was Administered in

14. Domain(s) of Social Cognition Investigated

15. Measurement Scale(s) or Instrument(s) Used

16. Key Findings

17. Conclusions

This content is neither created nor endorsed by Google.

Google Forms
