# Supplementary material for: Investigating the impact of psychedelic drugs on social cognition defects: A scoping review protocol
Source: PLoS One. 2024 Jul 29;19(7):e0307491. doi: 10.1371/journal.pone.0307491 (PMC11285908; doi:10.1371/journal.pone.0307491)
Supplement: S1 File — (DOCX) [file pone.0307491.s003.docx]

| Search # | Search Texts and Syntaxes | Results |
| --- | --- | --- |
| #1 | 3,4-methylenedioxyamphetamine/ or lysergic acid diethylamide/ or mescaline/ or n,n-dimethyltryptamine/ or n-methyl-3,4-methylenedioxyamphetamine/ or psilocybin/ | 11469 |
| #2 | (psychedelic* or "serotonin 2A receptor agonist*" or DMT or psilocyb* or psilocib* or ayahuasca or ayawasca or entactogen* or mdma or methylenedioxyamphetamine or ecstasy or mescalin* or magic mushroom* or psilocin or lysergic acid diethylamide or lsd or ghb or gamma- hydroxybutyrate or hallucinogen*).mp. | 30876 |
| #3 | ketamine.mp. | 24491 |
| #4 | N-methyl-D-aspartate antagonists.mp. | 240 |
| #5 | 1 or 2 or 3 or 4 | 54643 |
| #6 | ("social cognition" or "social process*" or "social function*" or "social* impair*" or "social* reject*" or "social communication" or "social review" or ((non-verbal* or non-facial* or facial*) adj3 communicat*) or RDoC or research-domain-criteria or autis* or facial expression* or reciprocat* or reciprocal or self-awareness or self-knowledge or interpersonal or empathy or "romantic relationships" or "intimate partners" or "sexual relationship*" or intimacy or "intimate relationship*" or "facial recognition" or (recogni* adj3 emotion*)).mp. | 341079 |
| #7 | 5 and 6 | 725 |

**Appendix I: Search strategy developed for PubMed for the scoping review on the impact of psychedelic drugs on social cognition defects in psychiatric and neurodevelopmental disorders (13th February 2024)**
