# Supplementary material for: Investigating the impact of psychedelic drugs on social cognition defects: A scoping review protocol
Source: PLoS One. 2024 Jul 29;19(7):e0307491. doi: 10.1371/journal.pone.0307491 (PMC11285908; doi:10.1371/journal.pone.0307491)
Supplement: Attachment — Submitted filename: Responses to Reviewers.docx [file pone.0307491.s004.docx]

Manuscript number: PONE-D-24-10240

Title: Investigating the impact of psychedelic drugs on social cognition defects: A scoping review protocol

Reviewer 1:

**Comment:** Smith et al. propose a way to investigate the research related to the use of psychedelics to improve social cognition skills. This is an extremely important aspect of psychedelic treatment and deserves the wide overview that they offer to present a summary of the literature.

**Author’s Response and Changes:** Thank you for your feedback.

**Comment:** The scoping review protocol that they propose, while limited to English language publications, focuses on social cognition defects in subjects with psychiatric diagnoses and neurodevelopmental disorders. Hopefully this will be a beginning protocol that can reveal the need for further research and possibilities for the role of psychedelics in mental health care. Furthermore, focused research will be necessary to hone the possibilities for a myriad of psychedelic substances, which vary tremendously in their effects.

**Author’s Responses and Changes:** Thank you for your feedback.

**Comment:** Smith et al. make no distinction between the different substances or the nonclinical contexts in which they are used, highlighting the need for research that specifies the set and setting, history, traditional uses and context for each substance. Each of these factors and several others greatly affect the consequences of psychedelic use. Other factors which must be queried include the prior experience of subjects with psychedelic experiences, their mental health care and context (including prior care, family history and aftercare) as well as their spiritual beliefs and experiences, and the community/cultural context within which the substances are used.

**Author’s Response and Changes:** Thank you for outlining the key factors that may affect users' perspectives and experiences of psychedelics, which will further impact social cognition. In our scoping review, addressing the limitation of not distinguishing between different substances and nonclinical contexts in psychedelic research requires a multifaceted approach:

Thank you for outlining the key factors that may affect users' perspectives and experiences of psychedelics, which will further impact social cognition. In our scoping review, addressing the limitation of not distinguishing between different substances and nonclinical contexts in psychedelic research requires a multifaceted approach:

The authors will pay attention to the set and setting in which each psychedelic drug is administered in included studies, taking note of factors such as the participant's mindset, expectations, and the physical environment where the experience takes place. Any provided information will be included in the scoping review. Furthermore, research will also be performed on the nonclinical contexts in which they are used

The authors will also conduct extensive research on each substance's historical and traditional context and include this information in the scoping review. Future clinical studies should, therefore, consider such information when designing their studies.

The authors will note any information provided on participants' prior experience with psychedelic drugs, mental health history, ongoing mental health care, spiritual beliefs, and cultural background and provide the information when discussing each included study. If information is not provided, it will be noted in the scoping review and mentioned that further studies should investigate these factors.

The scoping review will note any aftercare protocols followed in the included studies and stress their importance for maximizing benefits and minimizing adverse effects. Furthermore, any longitudinal studies that demonstrate the long-term effects and outcomes of psychedelic treatments will be discussed. If there is a lack of longitudinal studies, this will be noted, and the importance of such studies will be stressed.

Moreover, the importance of incorporating perspectives from psychology, neuroscience, anthropology and cultural studies in the investigation of psychedelics will be mentioned as a diverse perspective on psychedelic drugs is critical to understanding the many aspects that impact the usage and benefits of psychedelic drugs.

**Comment:** While Smith et al on the one hand, throw a wide net (including a vast array of psychedelics in their protocol), on the other hand they limit their protocol to studies with subjects who have defects in social functioning. This narrow focus ignores the enormous body of research that examines psychedelic experiences for wellness, enhancing mental health for those who are seeking spiritual expansion, and the ways psychedelic experiences are tools for enhanced social cognition and social cohesion outside of a clinical setting. However, if the protocol remains narrow it can illuminate gaps in research and a possible direction for future research and funding.

**Author’s Response and Changes**: We thank the reviewer for this insightful observation and comments. It is a hard balance to include all possible areas and multiple psychedelics in one scoping review. On the one hand, we are aware of the widespread impact psychedelics can have on the human body, mind and spirit; these findings are fascinating and critical for understanding the biopsychosocial influence of psychedelics; on the other hand, handling such a comprehensive topic is a challenging task and may easily dilute the focus and lose the target audience. Thus, we chose to structure our protocol to be useful to a certain audience (e.g., clinicians) and narrowed the social cognition domain that is mostly relevant to clinicians’ daily tasks. Furthermore, narrowing the protocol enables us to identify any positive or negative effects of psychedelics and gaps in the literature that would be specifically relevant to the clinical population, allowing our study to be the most beneficial it possibly can be to clinicians who work with patients with social cognition deficits.

Reviewer 2:

**Comment:** The proposal to prepare a scoping review that systematically illustrates the impact of psychedelic drugs on social cognition by Smith, Smith, Dennett, and Zhang has been carefully developed and will ultimately serve as a timely contribution within a rapidly emerging clinical & experimental space. I have no doubts that - in its final form - this review will provide an informative reference for a wide audience – in line with diverse readership at PLoS One.

**Author’s Response and Changes**: Thank you for your feedback

**Comment:** I very much appreciate the authors’ outlining of their PRISMA-ScR checklist. In addition, the question being asked is clear, the strategy to identify and include targeted literature appears unbiased and sound, and there is a consensus towards the plan to explore and report on the peer-reviewed & published data.

I have only three points that may increase the clarity and confidence of this Scoping Review’s message:

**Author’s Response and Changes:** Thank you for your feedback

**Comment:** First, I encourage the authors to emphasize effects of specific drugs in this class, and to include precise details about dose-effect outcomes; as the pharmacology of these drugs is often biphasic -with ascending and descending limbs on behavioral, cognitive, and emotional endpoints.

**Author’s Response and Changes:** The following has been added to Introduction (section Background) – line 65:

3-4- methylenedioxymethamphetamine (MDMA) has been shown to cause an enhanced mood, emotional warmth, empathy and a willingness to discuss emotionally charged memories [9]. Lysergic acid diethylamide (LSD) causes a feeling of closeness to others, openness, and enhanced emotional empathy [10]. Studies reveal that psilocybin enhances feelings of connectedness, increases openness, and increases altruism [11]. N, N-dimethyltryptamine (DMT) is known to create distortions in auditory and time perception and feelings of ego dissolution [12]. Several psychedelic drugs also have dose-effect outcomes, with high doses (≥ 3mg/kg) of MDMA producing antidepressant effects, some addictive potential and amnesia of fear conditioning memory, and low doses (≤ 1 mg/kg) not producing any of these effects [13]. Similarly, 5 mcg of LSD has been demonstrated to be the minimal dose required for observable effects. However, 20 mcg produces the most apparent effects [14].

In addition, I have also added the following references for this change:

NIDA. What are the effects of MDMA? . 13 Apr 2021 [cited 26 May 2024]. Available: https://nida.nih.gov/publications/research-reports/mdma-ecstasy-abuse/what-are-effects-mdma

Liechti ME. Modern Clinical Research on LSD. Neuropsychopharmacol. 2017;42: 2114–2127. doi:10.1038/npp.2017.86

Lowe H, Toyang N, Steele B, Valentine H, Grant J, Ali A, et al. The Therapeutic Potential of Psilocybin. Molecules. 2021;26: 2948. doi:10.3390/molecules26102948

Reckweg JT, Uthaug MV, Szabo A, Davis AK, Lancelotta R, Mason NL, et al. The clinical pharmacology and potential therapeutic applications of 5‐methoxy‐N,N‐dimethyltryptamine (5‐MeO‐DMT). Journal of Neurochemistry. 2022;162: 128–146. doi:10.1111/jnc.15587

Pantoni MM, Kim JL, Van Alstyne KR, Anagnostaras SG. MDMA and memory, addiction, and depression: dose-effect analysis. Psychopharmacology. 2022;239: 935–949. doi:10.1007/s00213-022-06086-9

Hutten NRPW, Mason NL, Dolder PC, Theunissen EL, Holze F, Liechti ME, et al. Mood and cognition after administration of low LSD doses in healthy volunteers: A placebo controlled dose-effect finding study. European Neuropsychopharmacology. 2020;41: 81–91. doi:10.1016/j.euroneuro.2020.10.002

**Comment:** Second, it will be useful to be sure an overview (perhaps additional Heading) of how any risks of bias are clearly avoided in the interpretation & consensus of meaning, as interpreted from study results. For example, I suspect highlighting discrepancies resulting from dose-related differences may bolster awareness of attention to crucial details in such a way as to avoid bias.

**Author’s Response and Changes:** The following has been added to Methods (section Data summarization and presentation) – line 190:

At this stage, risks of bias will be avoided by highlighting discrepancies that result from population, administration, or dose-related differences.

**Comment:** Finally, even though the Methods - under the Heading: Screening Strategy - mentions the use of two reviewers to verify inclusion and exclusion criteria, and even suggests a third, if necessary, I could argue there is utility in again identifying the use of two (or three reviewers) within the flow chart provided in the flow of Figure 1 (S1 Checklist).

**Author’s Response and Changes:** The following texts have been removed from Figure 1:

Records screened (n= )

Full-text articles assessed for eligibility (n= )

In their place, the following has been added:

Records screened by two reviewers (n= )

Full-text articles assessed for eligibility by two reviewers (n= )
